# Predicting the Incidence and Prognosis of Bone Metastatic Breast Cancer: A SEER-Based Observational Study

**DOI:** 10.1155/2020/1068202

**Published:** 2020-11-25

**Authors:** Dongning Shi, Junwen Bai, Yibo Chen, Xia Wang, Yafeng Zhang, Hong Liu

**Affiliations:** ^1^The Second Department of Breast Cancer, Tianjin Medical University Cancer Institute and Hospital, National Clinical Research Center for Cancer, Tianjin 300060, China; ^2^Key Laboratory of Cancer Prevention and Therapy, Tianjin 300060, China; ^3^Key Laboratory of Breast Cancer Prevention and Therapy, Tianjin's Clinical Research Center for Cancer, Tianjin Medical University, Ministry of Education, Tianjin 300060, China; ^4^Department of Surgery, Affiliated Hospital of Inner Mongolia Medical University, Hohhot, Inner Mongolia Autonomous Region 010050, China

## Abstract

**Background:**

To determinate the association relationship of breast cancer bone metastasis and cancer characteristics and molecular subtype. Furthermore, to evaluate the impact of molecular subtype on prevalence and prognosis of bone metastasis from the breast cancer base on a large population real-word program, the Surveillance, Epidemiology, and End Results (SEER) database.

**Methods:**

We collected and analyzed the data obtained from SEER, which showed molecular subtype information for each patient. The prevalence and outcome of bone metastasis in breast cancer were estimated as per the different molecular subtypes.

**Results:**

Occurrence of bone metastasis in conformity with four different molecular subtypes in all 42684 breast cancer patients was 6.2, 9.4, 7.9, and 6.4%, respectively. The most unfavorable subtype was the triple-negative breast cancer (TNBC), followed by the luminal A, luminal B, and HER2 subtypes (hazard ratio [HR] of luminal A compared with TNBC, 0.533, 95% confidence interval, 0.444–0.641; HR of luminal B, 0.482, 95% CI 0.419–0.555; HR of HER2 subtype, 0.542, 95% CI 0.484–0.608). Brain metastasis impacts overall survival (OS) (*p* < 0.001) fundamentally, and visceral metastases also significantly decreased OS (*p* < 0.001).

**Conclusion:**

Bone metastasis patients present a more favorable oncological survival consequence than other metastases, and the TNBC subtype with bone metastasis showed the poorest tumor outcome compared with the other three molecular subtypes.

## 1. Background

With the high incidence, breast cancer has become the most frequent malignant disease of women and the second most common cancer globally [1, 2]. Also, breast cancer had been the frequent main cause of all cancer deaths around the world. As we all know, the bone has been the most common site of distant metastasis and results in unfavorable outcomes [3], including some amount of complications like bone fracture, pain, and hypercalcemia, which can hardly be cured [4]. However, the blooming and implementation of hormone and targeted treatment for the breast cancer subtype have promoted greatly the oncological outcomes even for end-stage patients [5, 6]. In addition, various treatment modalities, including chemotherapy, hormone therapy, targeted therapy, immunotherapy, and radiopharmaceuticals can control cancer growth effectively and improve the quality of life by relieving symptoms like pain and constipation [7]. Therefore, predicting prognosis precisely of bone metastasis has become gradually urgent in choosing the most suitable therapy strategy.

Breast cancer molecular subtypes categorized as HER2 (HR−/HER2+), triple-negative breast cancer (TNBC, HR−/HER2−), luminal A (hormone receptor (HR+)/human epidermal growth factor receptor 2 (HER2−), and luminal B (HR+/HER2+), have been displayed empirically in clinical value in conducting therapeutic plans [8]. The choice of whether to execute HER2-targeted or hormone therapies is mainly dependent on the breast cancer molecular subtype. Previous researches have noted that the molecular classifications of breast cancer are meaningfully linked with risks of early tumor progression such as recurrence and metastasis [9], therapeutic response [10], and overall survival (OS) [11].

Our main objective of this study is to determine the association relationship of breast cancer bone metastasis and cancer characteristics, molecular subtype, and all other metastasis statuses and to value the effects of molecular types on prevalence and end results of bone metastatic breast cancer based on a large-scale population real-word database, the Surveillance, Epidemiology, and End Results (SEER) program.

## 2. Materials and Methods

### 2.1. Patient Selection

This study collected and analyzed the data obtained from the SEER latest release covering about 30% of the U.S. population in 2016. The details of each molecular subtypes were provided in the SEER-Medicare database. Moreover, the information was also obtainable regarding different metastasis containing bone, brain, lung, and liver at the first diagnosis of breast cancer.

Selection criteria of our study were shown as follows: (1) female breast cancer patients; (2) diagnosed year from 2011 to 2015; (3) age at diagnosis no less than 20 years old; (4) the information of breast cancer molecular subtype was available, and metastasis status existing in distant organs was known; and (5) breast cancer was first diagnosed or only cancer. Exclusive criteria were patients with untraced follow-up outcome information and diagnosed via autopsy or death certificate.

### 2.2. Statistical Analyses

Chi-square test was used to compare the incidence of bone metastasis among different subtypes. To predict the probability of bone metastasis more visibly, a nomogram was calculated and visualized. We separate randomly and evenly all patients into one training cohort and one validation cohort. Univariate and multivariate logistic regression analyses were implemented.

In subgroup analyses, including patients with stage IV cancer, bone metastasis, and each different molecular type, median overall survival (OS) of every group was evaluated by applying Kaplan-Meier estimates with log-rank tests. Of course, we also operate univariate and multivariate analyses to explore the risk factors of OS in these subgroups. We applied “Hmisc” and “rms” R packages to construct and visualize the nomogram of the prognosis prediction model with R software. In this study, we consider the two-sided *p* value less than 0.05 as statistically significant. All data were calculated using SPSS software (version 24.0, SPSS Inc., Chicago, IL, USA) and R studio platform under R 3.5.0 (http://www.r-project.org/).

## 3. Results

### 3.1. Bone Metastasis Rates Differ from Molecular Subtypes

Each breast cancer molecular subtype group in whole breast cancer patients were the following: luminal A, luminal B, HER2, and TNBC were 28931 (67.8%), 4502 (10.5%), 2244 (5.3%), and 7007 (16.4%), respectively. The incidence of bone metastasis in molecular subtype cohorts was 6.2, 9.4, 7.9, and 6.4% (Table 1). The luminal B and HER2 group have higher rates of bone metastasis. In younger patients with age less than 40 years old, the TNBC subtype presents even lower incidence of bone metastasis (4.9%).

Our data also showed that high clinical T and N stages were associated with a high prevalence of bone metastasis, while compared to other molecular subtypes, TNBC had a lower rate of bone metastasis relatively. In the brain only without the liver or lung metastasis group, the occurrence of bone metastasis in TNBC subtypes was lowest (13/49, 26.5%, *p* < 0.001). For the patients accompanied by liver or lung metastasis without brain metastasis, the HER2 and TNBC suggested lower frequencies of bone metastasis than the other two types. However, the percentage of bone metastasis had no significant difference in these four molecular subtypes (*p* = 0.958) in the group who had both brain and visceral metastases. If patients had none of the brain, liver, and lung metastases, the number of bone metastasis in both TNBC (191/6350, 3%) and HER2 (50/1959, 2.6%) patients was lower than that in luminal A (999/27734, 3.6%) and B (164/4074, 4%) subtypes (*p* = 0.003).

### 3.2. Nomogram Predictive Model

**Figure 1:**
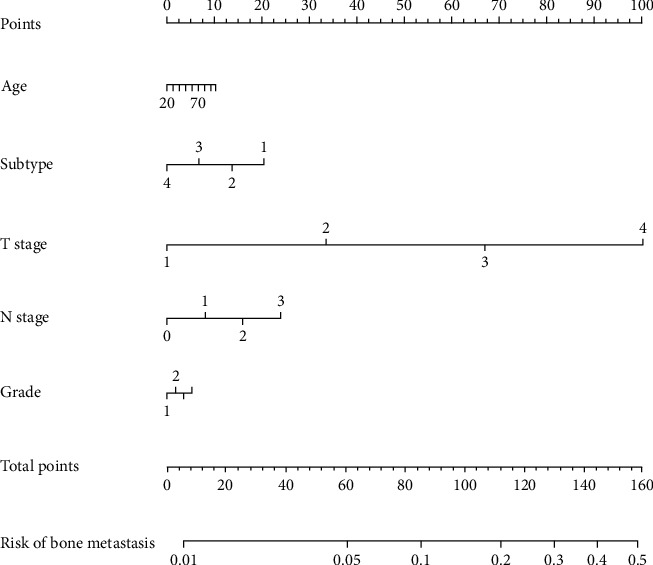
Nomogram predicting the probability of brain metastasis at the time of breast cancer diagnosis. Subtype: 1: luminal A; 2: luminal B; 3: HER2+; 4: TNBC. T stage: 1: ≤1.

Our multivariate regression found that the status of other metastases including brain, lung, and liver, older age at diagnosis, white and black race, high pathological grade, high clinical T and N stages, and luminal A was attributed to a high rate of bone metastasis (Table 2). To evaluate the trend of bone metastasis more precisely, we generated a nomogram base on the above logistic analysis (Figure 1). Based on the nomogram, if there is a breast cancer patient with 60 years of age, grade 3, T4N2, and luminal A, the tendency of bone metastasis is over 40% with a final total score of 148. Conversely, the occurrence of bone metastasis was nearly 10% at a lower level for patients aged 70 years, grade 3, T3, N2, and TNBC.

### 3.3. Prognosis of Bone Metastasis Patients in Subgroup Analysis

The overall survival time for stage IV cases was assessed depending on different molecular subtypes (Table 3). The median overall survival period was 18 months, 18 months, 11 months, and 9 months in the luminal A, luminal B, HER2, and TNBC group, respectively, revealing that the TNBC and HER2 cohorts featured the most disadvantageous survival outcome. For patients who had brain and lung metastases with or without liver metastasis, the prevalence of bone metastasis had no significant difference among varied molecular subtypes (*p* = 0.182 and *p* = 0.591, respectively). The HER2 and TNBC have shown a similar survival outcome, whether bone metastasis existed or not. With bone metastasis, however, unlike the HER2 subtype, the prognosis of luminal B patients was obviously better.

In the HER2 and TNBC group, patients in all age groups showed lower survival rates (Table 4), compared with the luminal A and B patients, and youth was related to a favorable prognosis. Tumor grade and T stage were not notable prognostic elements for bone metastasis except for the luminal A molecular subtype. Higher N stage was relevant to better prognosis in the luminal A and HER2 subtypes of bone metastatic breast cancer patients. In all four molecular subtypes, other site metastases were obviously related to the decreased median survival time of bone metastasis patients, especially in the brain metastasis subgroup. Meanwhile, brain and visceral metastases also correlated with survival time in TNBC and HER2 subtype patients.

### 3.4. Univariate and Multivariate Analyses

**Figure 2:**
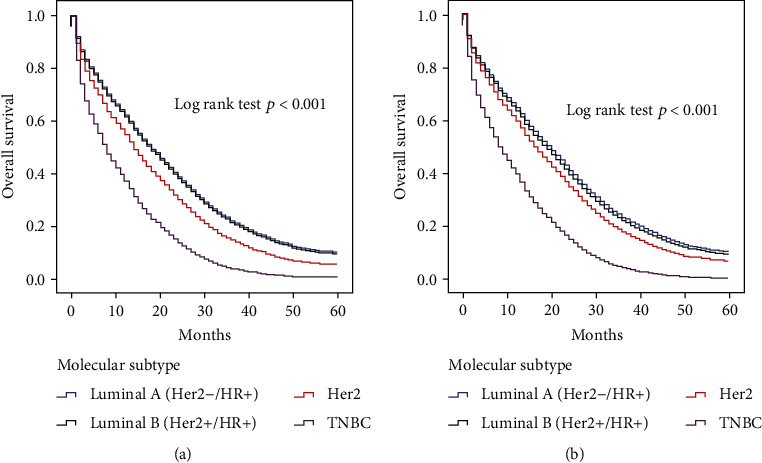
Kaplan-Meier survival curves according to molecular subtype: (a) in all patients with brain metastasis from breast cancer; (b) in patients with brain metastasis from breast cancer without brain metastasis.

In univariate analysis, variables including age, tumor grade, T stage, molecular subtypes, brain metastasis, liver metastasis, and lung metastasis were statistically significant (*p* < 0.05) for OS of bone metastatic breast cancer patients. So, we put all these variables into multivariate analysis. The molecular classification was considerably associated with OS (Table 5). Patients with the most unfavorable prognosis were the TNBC, poorer than luminal A, luminal B, and HER2 subtypes (hazard ratio [HR] of luminal A compared with TNBC, 0.533, 95% confidence interval, 0.444–0.641, *p* < 0.001; HR of luminal B, 0.482, 95% CI 0.419–0.555, *p* < 0.001; HR of HER2 subtype, 0.542, 95% CI 0.484–0.608, *p* < 0.001). Brain metastasis impacts OS (*p* < 0.001) fundamentally, and liver and lung metastases also significantly decreased OS (*p* < 0.001 and *p* < 0.001, respectively). Multivariate Cox regression analysis of OS in bone metastasis which had no brain metastases was also calculated. This analysis is taken in age at diagnosis, molecular classification, and liver or lung metastases. Similar results have been found in that the most inauspicious result was also the TNBC subtype, followed by the HER2, luminal A, and luminal B subtypes (HR of HER2 subtype, 0.484, 95% CI 0.395–0.593, *p* < 0.001; HR of luminal A compared with TNBC, 0.484, 95% CI 0.395–0.593, *p* < 0.001; HR of luminal B, 0.481, 95% CI 0.414–0.559, *p* < 0.001) (Table 6; Figure 2). High T stage and tumor grade and liver or lung metastases also related to the poor OS of bone metastatic breast cancer without brain metastasis (HR of T stage, 1.216, 95% CI 1.118-1.322; HR of tumor grade, 1.183, 95% CI 1.084-1.291; HR of liver metastasis, 1.688, 95% CI 1.536-1.855; and HR of lung metastasis, 1.228, 95% CI, 1.122-1.344).

## 4. Discussion

In our study of cancer patients from 2011 to 2015 (*N* = 42684), luminal B breast cancer displayed the highest occurrence of bone metastasis (9.4%, 422/4502), and even in elderly patients with luminal B molecular subtype, the rate of bone metastasis was over 10%. As the most common metastasis, bone metastasis could notably increase the risk of other metastases, especially brain metastasis [12–14]. The predictive nomogram allows us to assess bone metastasis's probability by molecular features and some clinical characteristics. This nomogram may be pretty useful due to the high occurrence of bone metastasis in breast cancer patients. The nomogram in our study estimates the prevalence of accompanying bone metastasis at the diagnostic time of breast cancer. Because of the nature of observed research, our nomogram could be more appropriate to make the decision to add a bone scan when the probability of bone metastasis is really high according to our nomogram.

Our results indicated that the incidence of bone metastasis in luminal A and B were apparently higher than that in HER2 and TNBC patients, which was consistent with previous research conducted by Xiao et al. [9]. Interestingly, with the age growth, the rate of bone metastasis in TNBC was also increased, while the incidence in other molecular subtypes was not changed visibly. Also, we noted that the rate of bone metastasis was seemingly increased in brain and visceral metastasis breast cancer patients compared with brain metastasis lacking visceral metastasis. While lung metastasis existed, the brain metastasis was associated with a low rate of bone metastasis. A previous study demonstrated a difference in time duration to distant recurrence [15, 16] largely. ER-negative tumor related to early recurrence, while ER-positive tumor is associated with a sustained low risk of more than five years. In agreement with our study, Xiao et al. [9]. found that TNBC had a higher rate of brain, liver, and lung metastases but a significantly lower rate of bone metastases than luminal A tumors.

A recent study conducted by Kono et al. showed that patients with bone-only first metastasis tend to have longer OS than patients with others-only first metastasis [17]. Similarly, in our research, we also demonstrated that the median survival of stage IV breast cancer was longer in the bone metastasis cohort than for the patient without bone metastasis, especially in luminal A and B subtypes. Patients with initial bone metastasis had a more favorable 5-year survival proportion than those with other metastases. Luminal A and luminal B patients had a propensity of bone metastasis and were related to better tumor outcomes of patients with initial bone metastasis.

Breast cancer cell transmission and final metastasis organs that grow into the distance—mainly bones, lung, and the brain—represent a significant clinical problem. The disease is incurable and is the main cause of death in the general population of most patients with TNBC. Metastatic spread of tumor cells is a highly complex but difficult to understand process, with many complicated biological processes, such as invasive, angiogenic, genetic, and epigenetic changes, tumor-interstitial interaction, basal permeation membrane, and some extravasation of cancer cells to the distal tissue [18]. However, disseminated cells are often in a new environment, and proapoptotic signals stay quiescent in long-term secondary organ latency, also known as dormancy [19, 20]. At this stage, breast cancer cells could hardly be detected and show resistance to chemotherapy [19]. This is still an important clinical issue, because patients are usually viewed as “survivors” and can progress to metastatic disease many years later. Disseminated tumor cells (DTCs) can enter into the secondary organs' sleep state by existing for an indefinite period of the proliferative cycle or by balancing the proliferation and apoptosis. The success of dormancy emergence results from further development of surviving DTCs by assembling molecular genetic changes allowing interaction with the tumor microenvironment [19]. By illuminating these features, patients with metastatic sites could well adapt to the host microenvironment and start the colonization. Although critical issues have been made, some specific problems need to be highlighted. Recent efforts focus on clarifying the role of key genes, potential molecular mechanisms, and effects of the signal pathway involved in fatal metastasis propagation. These studies are crucial for research progress on new effective treatment methods for antitumor metastasis in TNBC.

This study presents some vital significations for clinical practice. At first, the nomogram including breast cancer molecular subtypes can help clinicians to identify and predict patients at increased risk for bone metastasis at diagnosis. Second, the risk evaluation of molecular feature-based bone metastasis may play a crucial role in the age of precision medicine. Meanwhile, undeniably, our study has several limitations. First of all, our study is retrospective, so there is inevitably selection bias. Details of systemic therapy including targeted, hormone, chemical, and radiation therapy information is not available in our study. In the second place, the data of the sequence of different metastases during the follow-up time are not quite available yet due to the SEER program not supporting its related data. Last but not least, the specific location or number of bone and other metastases could not be acquired, which results in making it difficult to evaluate prognostic value.

## 5. Conclusions

The predictive model we constructed in our study could calculate the probability of bone metastasis of breast cancer at an initial diagnosis based on different molecular subtypes and other critical clinical characteristics. Patients with bone metastasis had a more favorable oncological survival than other metastases, and TNBC with bone metastasis patients showed the poorest tumor outcome compared with the other three molecular subtypes.

## Figures and Tables

**Table 1:** Incidence of bone metastasis in different breast cancer molecular subtypes.

Characteristics	Luminal A			Luminal B			HER2			TNBC			*p* value
	(HR+/HER2-)			(HR+/HER2+)			(HR-/HER2+)			(HR-/HER2-)			
	Bone	All	(%)	Bone	All	(%)	Bone	All	(%)	Bone	All	(%)	
Total	1800	28931	6.2	422	4502	9.4	178	2244	7.9	447	7007	6.4	<0.001
Age (yrs)													
<40	104	1229	8.5	34	410	8.3	15	178	8.4	32	653	4.9	0.035
40-59	687	11834	5.8	193	2184	8.8	83	1071	7.7	189	3253	5.8	<0.001
≥60	1009	15868	6.4	195	1908	10.2	80	995	8	226	3101	7.3	<0.001
T stage													
≤1	215	16607	1.3	49	1930	2.5	20	789	2.5	45	2217	2	<0.001
2	583	8515	6.8	127	1622	7.8	38	811	4.7	121	2870	4.2	<0.001
3	336	2059	16.3	79	470	16.8	29	263	11	85	912	9.3	<0.001
4	666	1750	38.1	167	480	34.8	91	381	23.9	196	1008	19.4	<0.001
N stage													
0	427	18074	2.4	94	2337	4	35	973	3.6	82	3512	2.3	<0.001
1	831	7329	11.3	203	1394	14.6	82	759	10.8	217	2084	10.4	0.001
2	254	2064	12.3	55	443	12.4	25	275	9.1	60	706	8.5	0.023
3	288	1464	19.7	70	328	21.3	36	237	15.2	88	705	12.5	<0.001
Other metastases													
Brain(+)lung(-)liver(-)	60	83	72.3	14	26	53.8	8	16	50	13	49	26.5	<0.001
Brain(-)lung(+)liver(-)	316	479	66	47	105	44.8	20	75	26.7	68	254	26.8	<0.001
Brain(-)lung(-)liver(+)	206	344	59.9	94	156	60.3	37	92	40.2	77	170	45.3	<0.001
Brain(+)lung(+)liver(-)	30	43	69.8	12	16	75	4	13	30.8	15	40	37.5	0.002
Brain(+)lung(-)liver(+)	16	21	76.2	8	9	88.9	5	10	50	9	12	75	0.264
Brain(-)lung(+)liver(+)	142	190	74.7	69	98	70.4	40	62	64.5	54	68	50	<0.001
Brain(+)lung(+)liver(+)	31	37	83.8	14	18	77.8	14	17	82.4	20	24	83.3	0.958
Brain(-)lung(-)liver(-)	999	27734	3.6	164	4074	4	50	1959	2.6	191	6350	3	0.003

HR: hormone receptor; HER2: human epidermal growth factor receptor 2; TNBC: triple-negative breast cancer.

**Table 2:** Multivariate logistic regression analysis predicting bone metastasis from breast cancer.

Characteristic	OR	95% CI	*p* value
Age	1.004	1.001-1.008	0.01
Race			
White	Reference		
Black	1.024	0.902-1.162	0.719
Other	0.785	0.655-0.942	0.009
Unknown	0.237	0.033-1.708	0.153
Grade			
1	Reference		
2	1.521	1.283-1.803	<0.001
3	1.101	0.921-1.318	0.291
4	1.486	0.856-2.577	0.159
T stage			
≤1	Reference		
2	3.02	2.62-3.482	<0.001
3	5.576	4.722-6.585	<0.001
4	10.355	8.816-12.163	<0.001
N stage			
0	Reference		
1	2.157	1.917-2.426	<0.001
2	1.833	1.562-2.150	<0.001
3	2.519	2.151-2.951	<0.001
Subtype			
Luminal A	Reference		
Luminal B	0.849	0.737-0.979	<0.024
Her2+	0.377	0.304-0.469	<0.001
TNBC	0.431	0.373-0.496	<0.001
Brain metastasis			
No	Reference		
Yes	6.436	4.987-8.306	<0.001
Liver metastasis			
No	Reference		
Yes	10.59	9.173-12.226	<0.001
Lung metastasis			
No	Reference		
Yes	6.31	5.508-7.229	<0.001

OR: odds ratio; CI: confidence interval.

**Table 3:** Median survival of stage IV patients by molecular subtype and bone metastasis.

Characteristics	All		Luminal A		Luminal B		HER2+		TNBC		*p* value
			(HR+/Her2-)		(HR+/Her2+)		(HR-/Her2+)		(HR-/Her2-)		
	Median	95% CI	Median	95% CI	Median	95% CI	Median	95% CI	Median	95% CI	
Stage IV											
No bone metastasis	12	10.883-13.117	14	11.969-16.031	15	10.293-19.707	11	8.419-13.581	9	7.598-10.402	<0.001
Bone metastasis	16	15.126-16.874	19	17.724-20.276	20	16.779-23.221	10	6.887-13.113	9	7.828-10.172	<0.001
Multiple metastases											
Brain(+)lung(-)liver(-)	8	5.127-10.873	9	4.942-13.058	13	0-31.739	10	6.080-13.92	6	4.05-7.95	0.028
Brain(-)lung(+)liver(-)	15	13.618-16.382	16	13.924-18.076	23	18.698-27.302	11	7.608-14.392	12	10.219-13.781	<0.001
Brain(-)lung(-)liver(+)	13	11.445-14.555	15	13.035-16.965	11	5.202-16.798	19	13.024-24.976	8	6.108-9.892	<0.001
Brain(+)lung(+)liver(-)	7	4.934-9.066	7	3.337-10.663	10	4.120-15.880	6	1.890-10.110	6	4.458-7.542	0.182
Brain(+)lung(-)liver(+)	7	4.352-9.648	7	4.009-9.991	17	2.391-31.609	7	2.445-11.555	3	0-6.395	0.011
Brain(-)lung(+)liver(+)	8	6.626-9.374	10	7.753-12.247	9	5.132-12.868	5	1.915-8.085	6	3.605-8.395	0.001
Brain(+)lung(+)liver(+)	4	2.876-5.124	4	2.537-5.463	7	4.934-9.066	2	0.674-3.326	4	0.173-7.827	0.591

**Table 4:** Median survival time of bone metastasis cancer patients according to molecular subtype and patient characteristics.

Characteristics	Luminal A		Luminal B		HER2+		TNBC		*p* value
	(HR+/Her2-)		(HR+/Her2+)		(HR-/Her2+)		(HR-/Her2-)		
	Median	95% CI	Median	95% CI	Median	95% CI	Median	95% CI	
All	19	17.724-20.276	20	16.779-23.221	10	6.887-13.113	9	7.828-10.172	<0.001
Age									
<40	27	21.289-32.711	25	20.429-29.571	19	9.532-28.468	13	9.832-16.168	<0.001
40-59	24	22.144-25.856	24	19.463-28.537	12	6.792-17.208	10	8.263-11.737	<0.001
≥60	15	13.553-16.447	14	8.137-19.863	6	2.017-9.983	7	5.530-8.470	<0.001
*p* value		<0.001		<0.001		0.177		0.003	
Race									
White	19	17.446-20.554	19	14.887-23.113	11	6.617-15.383	9	7.714-10.286	<0.001
Black	16	13.831-18.169	22	17.254-26.746	8	3.118-12.882	10	8.013-11.987	<0.001
Other	24	20.056-27.944	7	0-22.245	12	0-24.834	7	2.340-11.660	0.006
*p* value		<0.001		0.594		0.422		0.971	
Grade									
1-2	22	20.406-23.594	19	12.275-25.725	11	4.575-17.425	8	5.613-10.387	<0.001
3-4	16	14.645-17.355	20	16.340-23.660	10	6.405-13.595	9	7.754-10.246	<0.001
*p* value		<0.001		0.996		0.739		0.736	
T stage									
0-2	22	20.445-23.555	23	19.581-26.419	10	3.607-16.393	10	7.712-12.288	<0.001
3-4	17	15.409-18.591	17	11.878-22.122	10	6.933-13.067	9	7.671-10.329	<0.001
*p* value		<0.001		0.117		0.341		0.114	
N stage									
0-1	18	16.455-19.545	17	12.033-21.967	9	5.656-12.344	8	6.697-9.303	<0.001
2-3	21	18.792-23.208	22	18.577-25.423	16	8.355-23.645	10	8.013-11.987	<0.001
*p* value		0.042		0.577		0.038		0.487	
Brain metastasis									
No	21	19.732-22.268	21	18.027-23.973	12	7.380-16.620	9	7.731-10.269	<0.001
Yes	7	4.822-9.178	9	4.756-13.244	6	1.637-10.363	6	4.522-7.478	0.002
*p* value		<0.001		0.003		0.002		0.001	
Liver metastasis									
No	22	20.623-23.377	25	22.414-27.586	11	6.905-15.095	11	9.673-12.327	<0.001
Yes	11	9.013-12.987	9	4.945-13.055	9	3.404-14.596	6	4.623-7.377	<0.001
*p* value		<0.001		<0.001		0.008		<0.001	
Lung metastasis									
No	22	20.661-23.339	21	17.898-24.102	18	12.664-23.336	10	8.388-11.612	<0.001
Yes	12	10.108-13.892	14	7.818-20.182	5	2.403-7.597	7	4.663-9.337	<0.001
*p* value		<0.001		0.081		<0.001		0.002	

**Table 5:** Cox multivariate analysis of overall survival in bone metastasis from breast cancer.

Characteristics	HR	95% CI	*p* value
Age at diagnosis (continuous)	1.018	1.015-1.021	<0.001
Subtype			
TNBC	Reference		
Luminal A	0.533	0.444-0.641	<0.001
Luminal B	0.482	0.419-0.555	<0.001
HER2+	0.542	0.484-0.608	<0.001
Brain metastasis			
No	Reference		
Yes	1.686	1.479-1.922	<0.001
Liver metastasis			
No	Reference		
Yes	1.673	1.531-1.828	<0.001
Lung metastasis			
No	Reference		
Yes	1.236	1.135-1.345	<0.001
Grade			
1-2	Reference		
3-4	1.193	1.098-1.297	<0.001
T stage			
0-2	Reference		
3-4	1.169	1.079-1.266	<0.001

**Table 6:** Cox multivariate analysis of overall survival in bone metastasis from breast cancer without brain metastasis.

Characteristics	HR	95% CI	*p* value
Age at diagnosis (continuous)	1.018	1.015-021	<0.001
Subtype			
TNBC	Reference		
Luminal A	0.484	0.395-0.593	<0.001
Luminal B	0.481	0.414-0.559	<0.001
HER2+	0.484	0.395-0.593	<0.001
Liver metastasis			
No	Reference		
Yes	1.688	1.536-1.855	<0.001
Lung metastasis			
No	Reference		
Yes	1.228	1.122-1.344	<0.001
Grade			
1-2	Reference		
3-4	1.183	1.084-1.291	<0.001
T stage			
0-2	Reference		
3-4	1.216	1.118-1.322	<0.001

## Data Availability

The datasets generated and analyzed during the current study are available in the SEER database repository, https://seer.cancer.gov/.

## Abbreviations

SEER: Surveillance, Epidemiology, and End Results
HR: Hormone receptor
TNBC: Triple-negative breast cancer
OS: Overall survival
HR: Hazard ratio
HER2: Human epidermal growth factor receptor 2
DTC: Disseminated tumor cells.
